# An update on the therapeutic implications of long-chain acyl-coenzyme A synthetases in nervous system diseases

**DOI:** 10.3389/fnins.2022.1030512

**Published:** 2022-11-24

**Authors:** Zhimin Wu, Jun Sun, Zhi Liao, Jia Qiao, Chuan Chen, Cong Ling, Hui Wang

**Affiliations:** ^1^Department of Neurosurgery, The Third Affiliated Hospital, Sun Yat-sen University, Guangzhou, Guangdong, China; ^2^Department of Rehabilitation Medicine, The Third Affiliated Hospital, Sun Yat-sen University, Guangzhou, Guangdong, China

**Keywords:** long-chain acyl-coenzyme A synthetases (ACSLs), fatty acid (FA) metabolism, ferroptosis, nervous system diseases, targeted therapy

## Abstract

Long-chain acyl-coenzyme A synthetases (ACSLs) are a family of CoA synthetases that activate fatty acid (FA) with chain lengths of 12–20 carbon atoms by forming the acyl-AMP derivative in an isozyme-specific manner. This family mainly includes five members (ACSL1, ACSL3, ACSL4, ACSL5, and ACSL6), which are thought to have specific and different functions in FA metabolism and oxidative stress of mammals. Accumulating evidence shows that the dysfunction of ACSLs is likely to affect cell proliferation and lead to metabolic diseases in multiple organs and systems through different signaling pathways and molecular mechanisms. Hence, a central theme of this review is to emphasize the therapeutic implications of ACSLs in nervous system disorders.

## Introduction

Long-chain acyl-coenzyme A synthetases (ACSLs) are composed of a CoA synthetases family that activates fatty acid (FA) with chain lengths of 12–20 carbon atoms by forming the acyl-AMP derivative in an isozyme-specific manner. ACSLs identified in mammals mainly include five members—*ACSL1*, *ACSL3*, *ACSL4*, *ACSL5*, and *ACSL6* (*ACSL2* has been deleted because its cDNAs correspond to the same gene as *ACSL1*)—which encode the corresponding proteins and are essential for FA catabolism, *de novo* lipid synthesis, and remodeling of membranes (Soupene and Kuypers, 2008; Tang et al., 2018; Kuwata and Hara, 2019; Quan et al., 2021).

**FIGURE 1:**
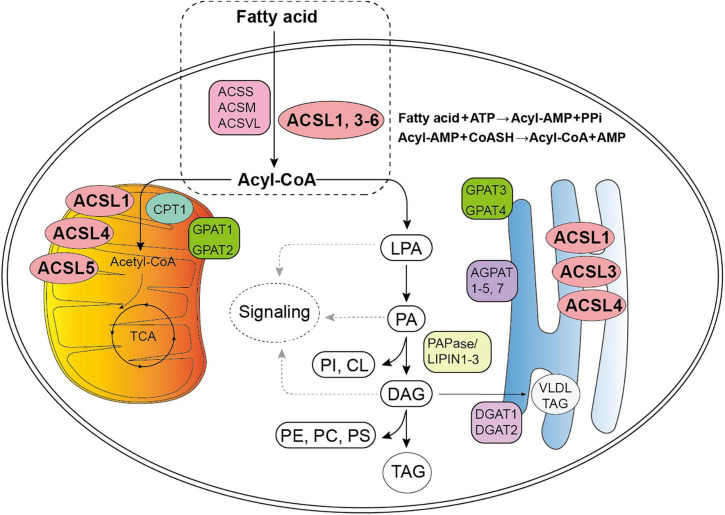
Diagram for fatty acid (FA) metabolism. The first stage of FA metabolism is the activation of FA, which requires a two-step reaction catalyzed by Acyl-CoA synthetases: an acyl-AMP intermediate is first formed from ATP, and then exchanged with CoA to yield the activated acyl-CoA. (Gassler et al., 2007; Soupene and Kuypers, 2008; Golej et al., 2011; Nakahara et al., 2012; Rossi Sebastiano and Konstantinidou, 2019). Subsequently, Acyl-CoA participates in the synthesis of lysophosphatidic acid (LPA), phosphatidic acid (PA), glycerol diester (DAG), and triacylglycerol (TAG) (Coleman, 2019). Acyl-CoAs can also be converted to acyl-carnitines by carnitine palmitoyltransferase (CPT1) to enter the mitochondria for β-oxidation and tricarboxylic acid cycle (TCA). The LPA, PA, and DAG intermediates may initiate signaling cascades, and PA and DAG are also precursors of all the glycerophospholipids: phosphatidylinositol (PI), phosphatidylcholine (PC), phosphatidylethanolamine (PE), phosphatidylserine (PS), and cardiolipin (CL) (Coleman, 2019). The TAG may remain in the cytosol within a lipid droplet or, in liver, be secreted a part of a very-low-density lipoprotein particle (VLDL) (Coleman, 2019). ACSS, short–chain acyl–CoA synthetase; ACSM, medium–chain acyl–CoA synthetase; ACSL, long–chain acyl–CoA synthetase; ACSVL, very long–chain acyl–CoA synthetase; AMP, adenosine monophosphate; PPi, pyrophosphoric acid; CoASH, coenzyme A; GPAT, glycerol-3-P acyltransferases; AGPAT, 1-acylglycerol-3-phosphate acyltransferases (also known as LPA acyltransferase); PAPase/Lipin, PA hosphohydrolases; DGAT, diacylglycerol acyltransferases.

It is well-known that FA metabolism is the major source of energy in mammals since it can release large quantities of adenosine triphosphate (Yan et al., 2015). The key step of long chain FA metabolism depends on its activation, which requires specific ACSLs (Figure 1). ACSLs have individual functions in FA metabolism among different types of cells, thus their dysregulation will contribute to a variety of metabolic diseases, such as fatty liver disease, obesity, atherosclerosis, diabetes, tumor, etc. (Yan et al., 2015). Several ACSLs-related reviews have summarized the mechanisms and function of the ACSLs in cancer: ACSL1 and ACSL3 may lead to cancer progression and worse prognosis, while ASCL5 and ACSL6 act as key suppressor with the opposite effect (Yan et al., 2015; Kuwata and Hara, 2019; Quan et al., 2021).

For the past decade, ACSLs have drawn the researchers’ attention to the areas of brain tumor (Yee et al., 2020), stroke (Chang et al., 2019; Chen J. et al., 2021; Cui et al., 2021), injury (Qu et al., 2021; Pang et al., 2022; Yuan et al., 2022), neurodegenerative disease (Yao et al., 2021; Ben-Zaken et al., 2022) etc. (Table 1). However, there has been no special review on the functions of ACSLs in nervous system diseases (NSDs) up to now. Here, we aim to highlight the important roles of ACSLs in NSDs.

**TABLE 1:** Long-chain acyl-coenzyme A synthetases (ACSLs) and nervous system diseases (NSDs).

	Genes*		Proteins*				
Name	OMIM^®^	Location	Size (Amino acids)	Molecular mass (Da)	Function	Nervous system diseases associated with ACSLs	
ACSL1	152,425	4q35.1	698	77,943	⊳ Catalyzes the conversion of long-chain FA to acyl-CoAs for both synthesis of cellular lipids ⊳ Preferred substrates: oleic acid and linoleic acid (Kanter et al., 2012)	*◇ Glioma* (Wang et al., 2018; Zhou et al., 2019; Xu et al., 2022) *◇ Amyotrophic lateral sclerosis (ALS)* (Ben-Zaken et al., 2022)	
ACSL3	602,371	2q36.1	720	80,420	⊳ Convert MUFAs (e.g.) into acyl-CoA that binds to membrane phospholipids ⊳ Preferred substrates: oleic acid, myristate, palmitate, arachidonate and eicosapentaenoate (Grevengoed et al., 2014)	*◇ Glioma* (Fujino et al., 1996, 1997; Van Horn et al., 2005; Qiu et al., 2020) *◇ Stroke* (Li et al., 2022)	
ACSL4	300,157	Xq23	711	79,188	⊳ Catalyzing PUFAs metabolism and shaping cellular lipid composition ⊳ Modulates glucose-stimulated insulin secretion by regulating the levels of unesterified epoxyeicosatrienoic acids ⊳ Modulates prostaglandin E2 secretion. ⊳ Preferred substrates: arachidonate (Klett et al., 2017)	◆Central nervous system *◇ Glioma* (Zhou et al., 2019; Cheng et al., 2020; Tan et al., 2020; Yee et al., 2020; Yi et al., 2020; Bao C. et al., 2021; Dattilo et al., 2021; Hacioglu and Kar, 2022; Kram et al., 2022; Miao et al., 2022) *◇ Cerebrovascular diseases: ischemic stroke* (Gubern et al., 2013; Li et al., 2019; Chen J. et al., 2021; Cui et al., 2021; Guo H. et al., 2021; Li C. et al., 2021; Liao et al., 2021; Hu et al., 2022; Tuo et al., 2022), *hemorrhage* (Chen B. et al., 2021; Jin et al., 2021), *subarachnoid hemorrhage* (Qu et al., 2021; Huang et al., 2022; Yuan et al., 2022) *◇ Injury: traumatic brain injury* (Kenny et al., 2019; Xiao et al., 2019; Bao Z. et al., 2021), *spinal cord injury* (Zhou et al., 2020; Pang et al., 2022) *◇ Intellectual disability: non-syndromic X-Linked intellectual developmental disorder* (Meloni et al., 2009; Zhang et al., 2009; Liu et al., 2011, 2014; Huang et al., 2016; Chang et al., 2019; Jia et al., 2019), *Alport syndrome with intellectual disability* (Rodriguez et al., 2010; Smetana et al., 2021) *◇ Neurodegenerative diseases: Alzheimer’s disease (AD)* (Rapoport, 2008; Thomas et al., 2017; Gao et al., 2021; Peng et al., 2021; Yan et al., 2022; Zhu et al., 2022), *Parkinson’s disease (PD)* (Li S. et al., 2021; Song et al., 2021) *◇ Cognitive dysfunction: diabetic cognitive impairment* (An et al., 2022), *postoperative cognitive dysfunction (POCD)* (Cheng et al., 2021) *◇ Others: epilepsy* (Kahn-Kirby et al., 2019; Mao et al., 2019; Shao et al., 2020, 2022; Yang et al., 2020; Chen et al., 2022), *ALS* (Moujalled et al., 2021; Zilka et al., 2021; Wang T. et al., 2022), *cerebral malaria* (Liang et al., 2022), *bipolar disorder (BD)* (Modi et al., 2014, 2017), *sepsis-associated encephalopathy (SAE)* (Wang J. et al., 2022)	◆Peripheral nervous system *◇ Neuropathic pain (NP) induced by peripheral nerve injury* <*ref*> (Zhang X. et al., 2022; Guo Y. et al., 2021; Wang et al., 2021)
ACSL5	605,677	10q25.2	683	75,991	⊳ Activates FA from exogenous sources for the synthesis of triacylglycerol destined for intracellular storage ⊳ Preferred substrates: palmitate, palmitoleate, oleate, linoleate (Klett et al., 2017)	*◇ Glioma* (Yamashita et al., 2000; Mashima et al., 2009a,b) *◇ ALS* (Iacoangeli et al., 2020; Nakamura et al., 2020; Saez-Atienzar et al., 2021)	
ACSL6	604,443	5q31	697	77,752	*◇* FA metabolism in brain *◇* Preferred substrates: It has equal preference for saturated and PUFAs with a backbone of C16–C20 (Lopes-Marques et al., 2013)	*◇ AD* (Pontifex et al., 2021) *◇ Schizophrenia* (Chen et al., 2006, 2011; Chowdari et al., 2007)	

ACSL, long-chain acyl-coenzyme A synthetase; FA, fatty acids; MUFAs, monounsaturated fatty acids; PUFAs, polyunsaturated fatty acids; OMIM, Online Mendelian Inheritance in Man. *Data from GeneCards (http://www.genecards.org/) and OMIM (https://omim.org/about).

### ACSL1

ACSL1 has a marked preference for oleate and linoleate (Kanter et al., 2012), and can promote ungoverned cell growth, facilitate tumor invasion and evade programmed cell death (Zhou et al., 2019; Xu et al., 2022). Several studies revealed expression of *ACSL1* is related to the progression and prognosis of glioma and amyotrophic lateral sclerosis (ALS).

#### Glioma

A previous analysis based on The Cancer Genome Atlas (TCGA) RNA sequencing data suggested lower expression of *ACSL1* influences metabolic reprogramming and contributes to the better survival of patients with isocitrate dehydrogenase 1 (IDH1) mutant glioma (Zhou et al., 2019). In a recent study on glioma, lower expression of *ACSL1* was also found to reverse the accelerated cellular metabolism and tumor growth induced by *PRADX* (a novel lncRNA ENST00000449248.1 identified by Xu et al., 2022) overexpression *in vivo* and *in vitro*. A total of six genes including *ACSL1* (the other five were *TGFBR2*, *RUNX1*, *PPARG*, *GIT2*, and *RAP1B*) have been characterized in glioma, which can interact with each other in both a competitive endogenous RNA-related manner and were predicted as markers of the mesenchymal subtype in terms of their protein functions (Wang et al., 2018). These findings provide some potential therapeutic targets for the treatment of human glioma.

#### Amyotrophic lateral sclerosis

ALS is a devastating progressive motor neuron disease that affects people of all ethnicities. A recent study on *ACSL1* found that A/G rs6552828 polymorphism may be associated with ALS, in which A-allele may be a risk factor for the development of ALS (Ben-Zaken et al., 2022). The authors analyzed at least 350 samples from 178 ALS patients and 172 athletes (including soccer players, middle- and long-distance runners) and found that the *ACSL1 AA* genotype was more prevalent among ALS patients and soccer players compared to controls, while *ACSL1 GG* carriers had a higher mortality rate (Ben-Zaken et al., 2022). This result suggests that ALS patients and soccer players may carry a common genetic predisposition, which is related to impaired FA utilization. However, given that little research on the connection between *ACSL1* and ALS has been published so far, more studies are needed to determine the regulatory mechanisms and therapeutic implications of *ACSL1* on ALS.

### ACSL3

The preferred substrates of ACSL3 are myristate, palmitate, arachidonate, and eicosapentaenoate (Grevengoed et al., 2014). As one of two predominant ACSL isoforms in the brain (another is ACSL6) (Van Horn et al., 2005), the expression of ACSL3 reaches a maximum level 15 days after birth, then declines gradually to 10% of the maximum in the adult brain (Fujino et al., 1996). Thus, ACSL3 may be closely related to the development of the brain.

#### Glioma

Fujino et al. (1997) found *ACSL3* existed in rat glioma cell line KEG1 cells two decades ago. Recently, Qiu et al. (2020) observed significant downregulation of *ACSL3* in U251 cells (human glioma cells) treated with 1.42 μM CN-3 (a new asterosaponin isolated from the starfish Culcita novaeguineae, which is characterized as exhibiting antitumor activities at low concentrations). It was reported that ACSL3 has relatively complex functions in different types of cancer. For example, its expression may vary in the different stages and types of cancer: ACSL3 was increased in early carcinogenesis to promote lipid anabolism and deposition, but deceased in advanced breast and prostate cancer to increase lipid utilization. Especially in breast cancer, ACSL3 was found to be upregulated in women with ER-negative breast cancer (Wang et al., 2013), while downregulated in triple-negative breast cancer (Wright et al., 2017). This difference and change in expression may be closely related to cancer cell survival and invasiveness (Tang et al., 2018). Although the similar finding has not been found in glioma till now, the *ACSL3* gene has been shown to be downregulated in U251 cells (as mentioned above) (Qiu et al., 2020). Future studies may discover more meaningful details about the changes in function of ACSL3 for glioma.

#### Stroke

A newly published article revealed that ACSL3 may play an important role in ferroptosis after cerebral I/R injury through GPX4/ACSL4/ACSL3 axis (Li et al., 2022). In transient middle cerebral artery occlusion (tMCAO) mice, the authors found that baicalein (an antioxidant from Scuetellaria baicalensis Georgi) could significantly increase the expression level of ACSL3, which suggested that ACSL3 is the negative regulator on ferroptosis (Li et al., 2022). The possible explanation may be that ACSL3 can convert monounsaturated fatty acids (MUFA) into acyl coenzyme A that binds to membrane phospholipids to protect cells from ferroptosis (Magtanong et al., 2019).

### ACSL4

**FIGURE 2:**
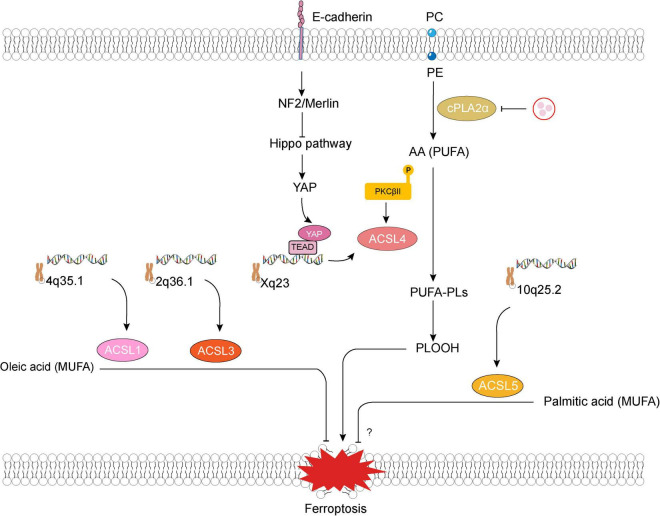
Long-chain acyl-coenzyme A synthetases family member 1, 3–5 (ACSL1, 3–5) and ferroptosis. E-cadherin–NF2–Hippo–YAP pathway suppresses ferroptosis by attenuating YAP-mediated transcription and translation of *ACSL4*. Lipogenesis involving production of phospholipids containing polyunsaturated fatty acid chains (PUFA-PLs) that are mediated by ACSL4 and multiple other enzymes is required for phospholipid peroxidation and ferroptosis (Jiang et al., 2021). Some pathological states (such as cerebral ischemia) would lead to an unexpected increase in thrombin within neurons, promote the mobilization of phosphatidylcholine (PC) and phosphatidylethanolamine (PE) in the phospholipid membrane of neuronal cells through cytosolic phospholipase A2alpha (cPLA2α), and accelerate the production of polyunsaturated fatty acid, such as arachidonic acid (AA) (Tuo et al., 2022). Besides, ferroptosis inducers promote a slight accumulation of lipid peroxide, which induces the activation of protein kinase C βII (PKCβII). Subsequently, activated PKCβII interacts directly with and phosphorylates ACSL4 at Thr328, which activates ACSL4, triggering the biosynthesis of PUFA-containing lipids and then promoting the generation of lipid-peroxidation products (Zhang H. L. et al., 2022). NF2, neurofibromin 2; YAP, Yes-associated protein; PLOOH, phospholipid hydroperoxide.

ACSL4 has a marked preference for arachidonate and plays an important role in neuronal differentiation in the brain (Cho, 2012; Klett et al., 2017). Knockout of *ACSL4* in embryonic stem cells markedly attenuated neuronal differentiation induced by all-trans retinoic acids and nerve growth factors (Cho, 2012). In the past decade, this enzyme has also been demonstrated essential for the induction of ferroptosis (a newly found form of programmed cell death) by facilitating arachidonic acid (AA) oxidation, which makes it a desirable target of some NSDs-related ferroptosis (Figure 2).

#### Glioma

Numerous glioma-associated studies have focused on the determination of ferroptosis-related protein expression, such as ACSL4, glutathione peroxidase (GPX4), system Xc^–^, and ferroptosis suppressor protein 1/AIFM2 (FSP1), etc. Mechanistically, ACSL4 is required for ferroptosis in glioma via the regulation of proliferation, migration of glioblastoma, and self-renewal of glia cells (Cheng et al., 2020; Bao C. et al., 2021; Dattilo et al., 2021). The decrease in the expression level of ACSL4 has been observed in isocitrate dehydrogenase (IDH) 1 wild-type and mutant gliomas (Zhou et al., 2019). The chemical inhibition, stable silencing, or depletion of ACSL4 demonstrated it can diminish necrosis and aggressiveness of glioblastoma (Yee et al., 2020). However, in the recurrent glioma, the expression of ACSL4 was found significantly increased, indicating glioblastoma relapses may be higher vulnerable to ferroptosis (Kram et al., 2022).

Subsequently, some targeting molecular mechanisms had been put up based on ACSL4, which shed light on the treatment of glioma. For example, miR-670-3p was found to suppress ferroptosis of human glioblastoma by inhibiting ACSL4, suggesting that inhibition of miR-670-3p can be an adjuvant strategy to treat glioblastoma (Bao C. et al., 2021). Dihydrotanshinone I (DHI) can inhibit the proliferation of human glioma cells via the induction of ferroptosis (Tan et al., 2020), while capsaicin can induce ferroptosis through the increase in expression of ACSL4 (Hacioglu and Kar, 2022). Besides, in erastin-induced ferroptosis in gliomas, heat shock protein 90 (Hsp90) and dynamin-related protein 1 (Drp1) were reported to actively stabilize and regulate ACSL4 expression. Hsp90 overexpression or calcineurin (CN)-mediated Drp1 dephosphorylation at serine 637 (Ser637) can promote ferroptosis via alteration of mitochondrial morphology and increase of ACSL4-mediated lipid peroxidation (Miao et al., 2022). These mechanisms might be used as potential anticancer agents or targets with ferroptosis-induced anti-proliferative effects.

#### Ischemic stroke

The increase of ACSL4 expression is frequently observed in the stroke models simulating ischemia/reperfusion neuronal injury (Li C. et al., 2021; Liao et al., 2021). Ischemia-induced ACSL4 activation can exacerbate ischemic stroke and contribute to ferroptosis-mediated tissue injury in ischemia/reperfusion (Cui et al., 2021). The up-regulation of ACSL4 may facilitate or hinder neurological recovery after stroke through the regulation of ferroptosis (Chen J. et al., 2021). Therefore, interventions of ACSL4 expression have been predicted to be the potential therapeutic target (Gubern et al., 2013). For example, a recent ferroptosis study pointed out that thrombin can induce ACSL4-dependent ferroptosis during cerebral ischemia/reperfusion, which suggested thrombin-ACSL4 axis may be an important therapeutic target to ameliorate ferroptotic neuronal injury during ischemic stroke (Tuo et al., 2022). Moreover, transcription factor special protein 1 (Sp1) has been identified as an important factor in promoting ACSL4 expression and exacerbating ferroptosis (Li et al., 2019). Pomelo peel essential oil (PPEO) also has a neuroprotective effect after cerebral ischemia/reperfusion injury by inhibiting the expression of ACSL4 and ferroptosis (Hu et al., 2022). Besides, carthamin yellow (CY) treatment can indirectly reverse ACSL4 expression level in the brain by the inhibition of Fe^2+^ and reactive oxygen species accumulation (Guo H. et al., 2021).

#### Intracerebral hemorrhage

Intracerebral hemorrhage (ICH) is one of the most refractory and lethal NSDs. Two ACSL4-associated mechanisms on ICH have been proposed, including lncRNA H19/miR-106b-5p/ACSL4 axis and HOTAIR/UPF1/ACSL4 axis (Chen B. et al., 2021; Jin et al., 2021), which may play a crucial role in ICH. In the first mechanism, miR-106b-5p is a target of H19, while *ACSL4* is a target gene of miR-106b-5p. This kind of regulation effect among H19, miR-106b-5p, and ACSL4 implicates ACSL4 may be downstream of this axis. With the use of the ICH model (glucose deprivation hemin-treated, OGD/H-treated), Chen B. et al. (2021) verified the knockdown of H19 can promote cell proliferation and suppress ferroptosis in the brain microvascular endothelial cells by regulating the lncRNA H19/miR-106b-5p/ACSL4 axis. Besides, HOTAIR/UPF1/ACSL4 axis has been also reported to play an important role in the ferroptosis of neuronal cells in the progression of ICH, which can be mediated by Paeonol (2′-hydroxy-4′-methoxyacetophenone), the main active compound of the radix of Paeonia suffruticosa (Jin et al., 2021). Paeonol notably inhibited ferroptosis in hemin-treated neuronal cells via inhibition of ACSL4. In short, the intervention of the axis may be a promising therapeutic strategy for ICH.

#### Subarachnoid hemorrhage

ACSL4 can exacerbate subarachnoid hemorrhage (SAH)-induced early brain injury (EBI) by mediating ferroptosis (Qu et al., 2021; Yuan et al., 2022). After SAH, the expression of ACSL4 in brain tissue increased significantly. Inhibiting the expression of ACSL4 using small interfering RNAs has been proven to alleviate inflammation, oxidative stress, brain edema, blood-brain barrier impairment, etc., and increase the number of surviving neurons (Qu et al., 2021). Furthermore, activation of SIRT1 (Sirtuin 1, a class III histone deacetylase) was found to suppress SAH-induced ferroptosis by deregulating the expression of ACSL4 (Yuan et al., 2022). Another study also demonstrated puerarin [8-(β-d-glucopyranosyl-daidzein)] can remarkably inhibit the expression of ACSL4 and ferroptosis, which is associated with EBI after SAH (Huang et al., 2022). The above evidence suggested ACSL4 could be a potential therapeutic target for SAH.

#### Traumatic brain injury

Increased expression of ACSL4 and other biomarkers of ferroptosis have been found in traumatic brain injury (TBI), which indicated ferroptosis is implicated in this pathological state and may contribute to neuronal death and worse functional outcome (Kenny et al., 2019; Xiao et al., 2019; Bao Z. et al., 2021). In a mice-based experimental study, the authors proved that cortical impact injury can result in accumulation of oxidized phosphatidylethanolamine, increased expression of 15-lipoxygenase and ACSL4, and depletion of glutathione in the ipsilateral cortex. These results can be reversed with the use of baicalein (12/15-lipoxygenase inhibitor) (Kenny et al., 2019). Another two mice-based studies also identified the increased expression of ACSL4 and the occurrence of ferroptosis in TBI, in which ferroptotic neuronal death can be attenuated by miR-212-5p and prokineticin-2 (Prok2) (Xiao et al., 2019; Bao Z. et al., 2021). Prokineticin-2 (Prok2) is an important secreted protein likely involved in the pathogenesis of TBI, which can suppress the biosynthesis of lipid peroxidation substrates, AA-phospholipids, via accelerated degradation of ACSL4 (Bao Z. et al., 2021).

#### Spinal cord injury

Edaravone, a lipid peroxidation scavenger, has been approved by Food and Drug Administration as a neuroprotective effect on spinal cord injury (SCI) and many other central nervous system diseases. It can downregulate pro-ferroptosis proteins ACSL4 and 5-lipoxygenase (5-LOX), and reduce microgliosis and astrogliosis to promote recovery after SCI (Pang et al., 2022). Proanthocyanidins (PACs) treatment has also been shown to mediate ferroptosis by significantly decreasing the levels of ACSL4 and iron in traumatic spinal cords and improving the locomotive function of SCI mice (Zhou et al., 2020). Lipoxin A4 (LXA4) can exert a protective role in SCI by inhibiting the expression of ferroptosis biomarkers including ACSL4 (Wei et al., 2021).

#### Non-syndromic X-Linked intellectual developmental disorder

Deletions or mutations of *ACSL4* are a rare cause of non-syndromic X-linked intellectual disability (Gazou et al., 2013). Zhang et al. (2009) and Jia et al. (2019) demonstrated that Drosophila ACSL-like protein, dAcsl, is highly homologous to human *ACSL3* and *ACSL4*; and the mutation of *dAcsl* can lead to the dramatical decrease of number of glial cells and neurons, which may further result in developmental defects. Liu et al. (2011) demonstrated that *dAcsl* can regulate axonal transport of synaptic vesicles and is required for synaptic development and function. Three years later, they further revealed that *dAcsl* can inhibit synapse growth by attenuating bone morphogenetic protein signaling via endocytic recycling (Liu et al., 2014). Another relevant study revealed that ACSL4 can inhibit neuromuscular junction growth by stimulating C16:1 fatty acyl production and concomitantly suppressing raft-associated lipid levels (Huang et al., 2016). Besides, Meloni et al. (2009) found the reduced levels of ACSL4 in the brain would induce a significant reduction in dendritic spine density and an alteration in spine/filopodia distribution. Chang et al. (2019) reported a very rare childhood stroke with *ACSL4* hemizygous intragenic deletion. These findings provide insights into the pathogenesis of *ACSL4*-related intellectual disability.

#### Alport syndrome with intellectual disability

Alport syndrome with intellectual disability (ATS-ID) is an X-linked contiguous gene deletion syndrome associated with an Xq22.3 locus, which is mainly characterized by neurodevelopmental disorder (NDD), hearing loss/deafness, hematuria, renal failure, midface retrusion, and elliptocytosis. It is thought that ATS-ID is caused by the loss of function of *ACSL4* genes through the interstitial (micro) deletion of chromosomal band Xq22.3 (Smetana et al., 2021). Another research reported a family with two males with this disorder, which is characterized by ID with absent or severely delayed speech, midface hypoplasia, and facial hypotonia (Rodriguez et al., 2010).

#### Alzheimer’s disease

Alzheimer’s disease (AD) is a progressive, age-related neurodegenerative disease. The expression of ACSL4 in the hippocampus in patients with AD has been shown to be related to dietary AA (Thomas et al., 2017). AA consumption is elevated in patients with AD, indicating an excess of AA in the human diet could constitute a risk factor for AD (Rapoport, 2008). In APP/PS1 mice (APPswe/PSEN1dE9 transgenic mice model of AD), transcriptome analysis identified the enriched ACSL4 (Yan et al., 2022), which can be inhibited by tetrahydroxy stilbene glycoside (TSG) (Gao et al., 2021). Besides, N2L, a novel lipoic acid-niacin dimer regulating lipid metabolism with multifunction, was also proved to exert neuroprotective effects by blocking the increase of ACSL4 protein expression (Peng et al., 2021). Interestingly, ACSL4 was also found to participate in AD-related cardiac contractile dysfunction, which can be rescued by mitochondrial aldehyde dehydrogenase (ALDH2) (Zhu et al., 2022).

#### Parkinson’s disease

Parkinson’s disease (PD) is another age-related degenerative brain disorder characterized by the loss of dopaminergic neurons in the substantia nigra and striatum. Recently, in 1-methyl-4-phenyl-1,2,3,6-tetrahydropyridine (MPTP)-induced PD mouse models, up-regulation of ACSL4 has been found and could be inhibited by apoferritin, a typical iron storage protein with a diameter of 12.5 nm. Apoferritin can improve motor deficits by preventing ferroptosis (Song et al., 2021). In another experiment stimulating cellular senescence, 1-methyl-4-phenylpyridinium (MPP) induced upregulation of ACSL4 expression and enhanced levels of oxidative stress, which were important characteristics of ferroptosis (Li S. et al., 2021).

#### Cognitive impairment

Diabetes has been shown to result in neurodegenerative diseases and cognitive decline, which can be alleviated by liraglutide (An et al., 2022). This drug mainly exerts its therapeutic effect by preventing the excessive amount of ACSL4 and inhibiting ferroptosis in the hippocampus (An et al., 2022). ACSL4 was also identified to be implicated in sevoflurane-induced postoperative cognitive dysfunction (POCD). In SH-SY5Y cells, increased ACSL4 expression can lead to ferroptotic neuronal death via the 5′ AMP-activated protein kinase/mammalian target of the rapamycin (AMPK/mTOR) pathway, while its downregulation has the opposite effect, providing a potential therapeutic approach to alleviate sev-induced POCD (Cheng et al., 2021).

#### Other central nervous system diseases

As ACSL4 dictates ferroptosis sensitivity via shaping cellular lipid composition, its inactivation has been considered a key mechanism for suppressing ferroptosis in diverse contexts (Doll et al., 2017). Ferroptosis has been shown to be involved in the neuronal damage, aberrant electrical brain activity (Shao et al., 2020, 2022; Chen et al., 2022), and the progressive death of motor neurons (Moujalled et al., 2021; Wang T. et al., 2022). Therefore, targeting ferroptosis-related protein ACSL4 may shed new light on the therapy of some other CNS diseases, including epilepsy (Kahn-Kirby et al., 2019; Mao et al., 2019; Shao et al., 2020, 2022; Yang et al., 2020; Chen et al., 2022), ALS (Moujalled et al., 2021; Wang T. et al., 2022), cerebral malaria (Liang et al., 2022), bipolar disorder (Modi et al., 2014, 2017), and sepsis-associated encephalopathy (Wang J. et al., 2022). For example, mood stabilizer valproic acid and chiral isomer valnoctamide have been shown to take effect in the treatment of bipolar disorder by inhibiting recombinant ACSL4, brain AA turnover in brain phospholipids, and AA activation to AA-CoA (Modi et al., 2014, 2017).

#### Neuropathic pain induced by peripheral nerve injury

Neuropathic pain (NP) induced by peripheral nerve injury has been shown to be associated with the over-expression of ACSL4 and ferroptosis (Guo Y. et al., 2021; Wang et al., 2021; Zhang X. et al., 2022). Here we introduce two identical NP models, including spared nerve injury (SNI) and chronic constriction injury (CCI). In the first rat models of NP, the expression of ACSL4 was found significantly increased in the spinal cord after SNI, which can be reversed by intrathecal injection of sirtuin 2 (SIRT2) overexpressed recombinant adenovirus, indicating that SIRT2 may achieve a neuroprotective effect via the suppression of ferroptosis (Zhang X. et al., 2022). In another CCI model of the sciatic nerve, Guo H. et al. (2021) also observed a similar phenomenon: the level of ACSL4 significantly increased in the corresponding spinal cord segment after injury. They further revealed that spinal ferroptosis-like cell death was involved in the development of NP resulting from injury, and inhibition of ferroptosis could alleviate mechanical and thermal hypersensitivities (Guo Y. et al., 2021). However, they failed to further reveal the inner molecule mechanism that affected ferroptosis, and Schwann cells (SCs), the basic cells of the myelin sheath of the axis cylinder, were not mentioned.

Several SCs-related studies have tried to clarify the mechanism of ferroptosis in peripheral nerve injury (Chang et al., 2021; Gao et al., 2022). Chang et al. (2021) pointed out complex IV subunit 4 isoform 2 (Cox4i2) can trigger an increase in reactive oxygen species, leading to ferroptosis and apoptosis in human herpesvirus 7 (HHV7) infected SCs. Gao et al. (2022) found the overexpression of c-Jun, a key regulator of the response of SCs to peripheral nerve injury, inhibits erastin-induced ferroptosis in SCs and promotes repair of facial nerve function.

Previous analysis of ferroptosis-resistant cell lines and a CRISPR suppression screen independently yielded ACSL4 inactivation as a key mechanism for suppressing ferroptosis in diverse contexts (Doll et al., 2017). ACSL4 may be more akin to caspase-3, the executioner of apoptosis, than to a housekeeping protein (Stockwell, 2022). These results implied there may exist a close relationship between ACSL4 and ferroptosis in the SCs, although changes in the expression of ACSL4 were not detected directly in the above two studies.

### ACSL5

ACSL5 is encoded by *ACSL5* gene, of which the preferred substrates are palmitate, palmitoleate, oleate, and linoleate (Klett et al., 2017). The current studies mainly reported its correlation with glioma and ALS.

#### Glioma

Mashima et al. (2009b) reported that ACSL5 is overexpressed in malignant glioma, and can selectively enhance human glioma cell survival through its ACS catalytic activity under extracellular acidosis. cDNA microarray analysis further suggested that *ACSL5* was critical to the expression of tumor-related factors including midkine (MDK), and the knockdown of MDK expression significantly attenuated ACSL5-mediated survival under an acidic state (Mashima et al., 2009b). Mashima et al. (2009a) also found that ACSL5 inhibition can synergistically potentiate the glioma cell death induced by etoposide, a well-known activator of apoptosis. In another earlier study, Yamashita et al. (2000) found FA-induced glioma cell growth is mediated by the *ACSL5* gene located on chromosome 10q25.1-q25.2, where deletion frequently happened in malignant gliomas. This evidence demonstrated the important role of ACSL5 on glioma cell growth.

#### Amyotrophic lateral sclerosis

In a study on genome-wide data analysis, *ACSL5* was identified as one of six differentially expressed genes through two-sample Mendelian randomization (Saez-Atienzar et al., 2021). Another genome-wide meta-analysis on data from European and Chinese populations (84,694 individuals) also found the *ACSL5*-ZDHHC6 locus is associated with ALS and links weight loss to the disease genetics—body weight loss is a frequent complication in ALS patients and is reported to be associated with shorter survival (Iacoangeli et al., 2020). This connection between *ACSL5* and ALS was also confirmed by Nakamura et al. (2020) in a genome-wide association study using 1,173 sporadic ALS cases and 8,925 controls in a Japanese population.

### ACSL6

ACSL6 is reported to have an equal preference for saturated and polyunsaturated FA with a backbone of C16–C20 (Lopes-Marques et al., 2013). ACSL6 is enriched in the brain and highly critical for maintaining brain omega-3 FA docosahexaenoic acid (DHA) levels (Chouinard-Watkins and Bazinet, 2018; Fernandez et al., 2018). DHA is also abundant in the brain and helpful in protection against numerous neurological disorders, and this type of protective effect can be enhanced by ACSL6 (Fernandez et al., 2018). However, it is noteworthy that the neuroprotection effect was confirmed to be confined only to neurons, not in astrocytes (Fernandez et al., 2021). Besides, ACSL6-related NSDs mainly include AD and schizophrenia.

#### Alzheimer’s disease

As one of the DHA transporters (another known is Fatp4), the alteration in the expression of ACSL6 may be a risk factor for an exacerbation of cognitive and neurological deficits in patients with AD (Pontifex et al., 2021). It was reported that overexpression of ACSL6 in nerve cells can significantly increase DHA and AA internalization within the first 24 h of neuronal differentiation to stimulate and enhance phospholipids synthesis and neurite outgrowth (Marszalek et al., 2005). Silencing ACSL6 inhibits axon outgrowth of mouse neural cells. ACSL6-induced activation of acetylcholinesterase may be involved in this process, as acetylcholinesterase promotes neural differentiation. The insufficiency of ACSL6 can lead to neuronal degeneration, while its over-expression is closely associated with neurite outgrowth (Kim et al., 2009). Lipid profiling of *ACSL6*^–/–^ (loss of *ACSL6*) tissues reveals consistent reductions in DHA-containing lipids in tissues highly abundant with ACSL6 (Fernandez et al., 2018), and *ACSL6*^–/–^ depletes brain membrane phospholipid DHA levels, which is related to motor function, memory, and age-related neuroinflammation (Fernandez et al., 2021).

#### Schizophrenia

*ACSL6* gene is also reported to be associated with schizophrenia (Chen et al., 2006, 2011). Its variation may contribute to the number of cigarettes smoked in patients (Chen et al., 2011). For example, nicotine exposure can stimulate the expression of ACSL6 in the prefrontal cortex and hippocampus of mice (*in vivo*), which can be suppressed by injection of the nicotinic receptor antagonist mecamylamine (Chen et al., 2011). However, in another candidate gene association analysis, the authors claimed their results did not yield convincing evidence for associations of schizophrenia with ACSL6 (Chowdari et al., 2007).

## Conclusion

ACSLs are involved in some biological responses by activating long-chain FAs in the nervous system, such as tumor development, progression and cell death. Several diseases are related to more than one subtype. For example, the progression of glioma is regulated by four members of ACSLs, including ACSL1, 3, 4, and 5; ALS is associated with the dysregulation of at least three subtypes of ACSLs, including ACSL1, 4, and 5.

However, most of the previous studies mainly focused on the central nervous system and presented encouraging results, while little evidence on the relationship between ACSLs and peripheral nervous diseases has been provided. Although some recent studies have proposed the key roles of ACSL4 in the mechanism of neuropathic pain induced by peripheral nerve injury, few studies are involved in the regulation effect of ACSLs in the SCs.

Furthermore, ACSL4 is universal in numerous NSDs because it correlates with ferroptosis and has been predicted to be the potential therapeutic target in some NSDs. It is noteworthy that ferroptosis was not mentioned in studies that suggested *ACSL4* gene dysfunction leads to intellectual disability. However, this doesn’t mean ferroptosis is not relevant to these diseases due to its close relationship with ACSL4. Further studies are needed to verify the inner correlation between ferroptosis and these neurological disorders.

What’s more, ACSL1, 3, and 5 are also reported to be ferroptosis-relevant isoforms. For example, ACSL1 can mediate α-eleostearic acid (ESA) -triggered ferroptosis as well as αESA incorporation into specific lipid species including DAGs and TAGs. ACSL3 can activate MUFAs (e.g., oleic acid) and promote a ferroptosis-resistant cell state. *ACSL5* has also been identified as the ferroptosis-related gene in cancer. Although they play important roles in non-NSDs, their functions in the nervous system have not been well proven.

In conclusion, plenty of encouraging findings indicated that targeting ACSLs and ferroptosis may be a novel potential therapeutic strategy, especially in the situation of NSDs.

## Author contributions

All authors listed have made a substantial, direct, and intellectual contribution to the work, and approved it for publication.
